# AttnEffNet-B4: an attention-augmented EfficientNet-B4 framework with fourier transformation for robust multi-disease diagnosis

**DOI:** 10.1038/s41598-026-49257-w

**Published:** 2026-04-18

**Authors:** Puneet Kumar, Deepika Kumar, Abhishek Kumar, Pramod Singh Rathore

**Affiliations:** 1 School of Engineering and Technology, CGC University, Mohali, 140307 Punjab India; 2 Department of CSE, Chandigarh University, Mohali, Punjab India; 3 Department of Computer and Communication Engineering, Manipal University Jaipur, Jaipur, India

**Keywords:** Disease diagnosis, Multi-disease, Healthcare, Self-attention, Deep learning, EfficientNet B4, Multimodality, Computational biology and bioinformatics, Diseases, Engineering, Health care, Mathematics and computing, Medical research

## Abstract

Early and precise disease detection in healthcare plays a vital role for better treatment results, healthier life expectancy, and good quality of care. However, many conventional AI diagnostic models are designed with a narrow focus on a single disease identification. This would limit their applications in the current-day healthcare system, particularly in remote and rural areas where there is limited access to specialized healthcare services. For the automated diagnosis, the study proposed a unified advanced multi-disease classification system, namely Attention Augmented EfficientNet-B4 (AttnEffNet-B4), incorporating Fourier transformation, a stratified cross-validation framework, and transfer learning with an attention technique. Various pre-trained convolutional neural networks, such as EfficientNetB0, VGG-16, VGG-19, ResNet-50, and InceptionV3, are also used in the study to evaluate a variety of medical imaging modalities, including chest X-rays, MRI scans, CT scans, and skin lesion images. To improve classification accuracy across a range of disorders, such as neurological, respiratory, dermatological, and ocular conditions, advanced preprocessing and feature extraction techniques are used in the proposed AttnEffNet-B4 approach. Experimental results show that the proposed model outperforms the various pre-trained approaches, reaching a peak accuracy of 97.69% on the training set and 94.47% on the testing set, which comprises multiple diseases. To validate the effectiveness of the suggested AttnEffNet-B4 model, a comparative analysis is further done with several pre-trained CNN models, followed by the stratified k-fold cross-validation. This work incites sustainable development goals for good health and well-being by providing early detection of diseases, access to health care, and reducing diagnostic errors through AI-based multi-disease classification.

## Introduction

In today’s era, as our lifestyles and living environments have changed, the prevalence of diseases such as diabetes, heart disease, skin disease, cancer, etc. has increased significantly. The year 2022 revealed the estimation of the global cancer burden in a report titled “*A Cancer Journal for Clinicians*” by the International Agency for Research on Cancer (IARC) and the American Cancer Society (ACS)^1^. According to the report, as per the public estimates, someday by 2050, the new cases of cancer every year could reach up to thirty-five million, thus increasing by 77% from figures reported in 2022. The report also highlights variations in cancer incidence according to human development levels in different parts of the world. Further, as per the report, among all types of cancer, lung cancer was the most prevalent one with around 2.5 million new cases, or one in every eight cancers globally (12.4% of all). Breast cancer (11.6%), mammary cancer (9.6% and colon cancer (9.3%) were the other most commonly diagnosed cancers. The report estimated almost 1.8 million deaths from lung cancer, making this type the most common cause of cancer-related deaths^2^. Another leading cause of death is heart attacks and strokes, which account for 85% of deaths from cardiovascular disorders globally^3^. This leading cause of non-communicable disease recorded the highest number of deaths in 2019. In terms of disability-adjusted life years (DALYs), stroke was the world’s second-greatest cause of death and the third-leading cause of both death and disability. The economic burden of stroke is estimated to exceed 721 billion dollars globally, accounting for 0.66% of the world’s GDP^4^. Other than Cardiovascular disorders, other forms of neurological illnesses are also a major health challenge. These are conditions, both congenital and neurodevelopmental, and those that can be neurodevelopmental and also neurological disorders that can impair individuals throughout their life span. Today, it has been found that more than 55 million individuals in the world have dementia, with over 60% living in low and middle-income nations. Dementia is caused by various brain-related injuries and diseases, with the most prevalent of them being Alzheimer’s, causing 60–70% of the cases^3^. Dementia has become the seventh leading cause of death and one of the most common causes of disability and dependency among the elderly across the globe. New cases are estimated at close to 10 million per year.

However, diseases related to the brain are not the only dominant health concerns. Skin conditions are believed to impact approximately 1.8 billion individuals at any given moment^4^. In regions with limited resources and warm climates, skin infections caused by bacteria, viruses, fungi, or parasites are the most prevalent source of illness. In many communities, skin conditions account for approximately 10% of all reported diseases^5^. Similarly, vision impairment is another widespread health issue, affecting approximately 2.2 billion individuals worldwide^6^. Vision impairment and blindness can impact individuals of any age, but the majority of people suffering from these conditions are over the age of 50, and the main factors contributing to vision impairment and blindness are refractive errors and cataracts. It is estimated that globally, only 36% of individuals with a vision impairment caused by refractive error and 17% of those with vision impairment due to cataracts have received access to a suitable treatment^7,8^. Therefore, as per the doctors and researchers, if these ocular diseases are diagnosed in an early stage and provided timely treatment, over one billion vision impairments can be prevented or treated. But, the financial impact of vision impairment is significant, with the global cost of productivity loss due to vision impairment estimated to be around US $411 billion annually^9^. Similarly, the increasing burden of other chronic diseases, including heart-related conditions, neurological disorders, skin diseases, vision impairments, etc., is also putting enormous pressure on health systems worldwide. Additionally, in an environment where medical resources are already limited, modern lifestyles that include sedentary behavior, poor dietary choices, and environmental pollution are increasingly being connected to almost all chronic diseases, the need for effective predictive tools is greater than ever^10,11^. To enhance the global health outcomes, all these new challenges have to be resolved with innovative and sustainable solutions. The emergence of this mounting crisis is causing a rethinking of the old disease diagnosis systems and treatment programs.

The emergence of Artificial Intelligence (AI) has rendered computers to now process information, analyze logically, make intelligent decisions, and act like humans^12^. AI is integrated with Machine Learning (ML), computer vision, Deep Learning (DL), and natural language processing, among others. ML algorithms use a collection of optimizations, statistical, and performance techniques to derive useful knowledge from the data and to come up with proper decisions. DL deals with big and complex data, identifying complex patterns and relationships that could further support early intervention and better prevention of diseases. Unlike ML, which relies on learning methodologies, DL incorporates multilayer neural networks that enable machines to interact within human-centric situations, making it a more advanced subset of ML^13,14^–^15^. Similarly, the healthcare industry has also seen significant advancements in AI, ML, and DL, especially in medical imaging, which has improved the capacity of medical personnel to identify and treat illnesses^16^. As much recent interest has been raised in AI, ML, and DL for their promise of enabling the improvement of disease diagnosis, numerous changes have also been reported in the diagnosis of disease due to these technologies. For example, the earlier systems mainly focused on detecting only one disease at a time. Initially, an AI and ML empowered model was made to diagnose a given disease with high accuracy using data sets designed specifically for one individual’s health concerns at a time. It has its benefits, but it is ineffective in a current real-world scenario, as patients often have multiple health problems. Hence, current developments attempt to introduce more versatile systems for diagnosing concurrent multiple health conditions by introducing a multi-disease identification framework. Here, label classification techniques and huge heterogeneous datasets help improve the diagnostics in terms of accuracy and efficiency. This development contributes to the clinical process as well as improves personalized health care since a patient will receive a more comprehensive overview of their overall health rather than just one disease diagnosis. In addition to it, early disease detection by using an advanced multi-disease framework is also impactful for the reduction of overall mortality rates.

The summary of the significance of Multi-Disease (MltD) detection in healthcare is as follows:

1. In modern health care, those who are going to have a surgery must consult a number of experts, including a dermatologist, pulmonologist, neurologist, and countless others. This kind of procedure typically includes various types of diagnostic examinations and imaging that require the patient to visit the clinic many times and stay in line. Such fragmentation and inefficiency are highly inconveniencing to the patients and a waste of resources, and slow down the process of scheduling surgeries among the service providers. Moreover, traditional models diagnose only one disease at a time. If separate models are employed for every disease, then it hinders the scalability of the same, increases the complexity of the system, cost of infrastructure and heavy computational resources would be needed to operate in a systematic way in the clinical workflow. All these obstacles can be overcome with the help of the MltD diagnostic model, leading to a sustainable solution to enhance global health outcomes.
2. Regular health checks typically involve various tests on patients for many diseases, such as tests for heart-related diseases (ECG and X-rays), skin check-ups for cancers, and the health of the brain through MRI or CT scans. These investigations could be analyzed together in an MltD diagnostic approach that utilizes all the images and results generated in the tests mentioned above into a single detailed report, thereby saving time and increasing the probability of early detection.
3. In rural and remote areas where access to specialists is limited, healthcare providers with more basic training can use telemedicine-assisted MltD diagnostic systems to capture medical images (lung X-rays, skin lesion photos, and head scans) and receive multi-condition diagnostic results from an intelligent model. This minimizes the need for patients to travel to remote hospitals, thus guaranteeing rapid medical interventions. Similarly, rapid decision-making is a must in emergency rooms, like a patient who arrives with various signs and symptoms, i.e., shortness of breath, skin rashes, or one that may be an indicator of a stroke, needs urgent evaluations. Therefore, MltD diagnostic models can assist in the rapid identification of multiple conditions by analyzing several medical images and assist in faster and more accurate triaging decision-making.

The contribution of the presented study is summarized as follows:

1. Propose a novel AttnEffNet-B4 that augments the EfficientNet-B4 backbone with a self-attention mechanism that applies channel attention to reweight the feature maps and spatial attention to highlight clinically relevant regions.
2. Integrate a comprehensive set of image enhancement approaches, including Fourier Transformation to work in the frequency domain for the analysis of features, CLAHE for local contrast enhancement, Gaussian noise to enhance robustness, and numerous data augmentation techniques to further aid in generalization across heterogeneous medical modalities.
3. Employ a stratified five-fold cross-validation to attain an unbiased and reliable performance measure estimation on the imbalanced multi-class dataset for real-world clinical deployment.

In summary, MltD diagnostic models have a lot of benefits, such as increased efficiency, availability, diagnostic precision, and decreased reliance on expert consultations. Therefore, this study presents an AttnEffNet-B4 for MltD diagnosis. Four different diseases affecting the skin, brain, eye, and lung, with a further twenty-eight sub-classifications, have been chosen for precise prediction. The proposed AttnEffNet-B4 is also compared to the state-of-the-art models that are currently in existence, and a stratified five-fold cross-validation is conducted, demonstrating its usefulness and its potential in improving healthcare diagnostics. The paper is also broken down to include sections, including the related work, methodological workflow, results, discussion, conclusion, and futuristic approaches.

## Related work

The development of AI and ML has transformed the healthcare sector and contributed to the accuracy, efficiency, and scalability. The conventional diagnostic approaches that primarily involve manual diagnosis and the concept of a single disease cannot meet the growing requirement of intricate disease diagnosis and timeliness. MltD diagnosis is one of the main fields of research that requires one to diagnose and classify many diseases simultaneously. In order to improve the diagnostic performance, recent researches have paid attention to ensemble learning algorithms, DL models, and multi-modal data fusion. Nevertheless, some studies also include information heterogeneity, model explainability, and their practical application in clinical settings. The section offers the perspectives of the current and future trends of AI-assisted MltD diagnosis, as well as an overview of paradigm shifts, the present-day methodologies, and gaps in the literature.

Yaganteeswarudu et al.^17^ proposed a prediction system of MltD using the Flask API, ML algorithms, and TensorFlow for the prediction of diseases, like Diabetes, Diabetes Retinopathy, Heart Disease, and Breast Cancer. In the model suggested by Arumugam et al.^18^, the Cleveland dataset used was fed to a preprocessor to remove noise and maintain data consistency for further cleansing. Final processed and consistent data is subjected to ML classification, such as Support Vector Machines, Naive Bayes, and Decision Tree C4.5. Gupta and Singh^19^ came up with an intelligent MltD diagnosis architecture based on UCI repository data. They compared the AdaBoost ensemble classifier coupled with other ML algorithms and found that it offered the best classification accuracy for the Cleveland Pima dataset. While the proposed architecture exhibits scope for improvement in terms of time complexity, it has the potential to classify various other life-threatening diseases in addition to those examined in this study.

Ampavathi et al.^20^ created a MltD prediction model using an enhanced DL approach. They collect datasets on various diseases from the UCI repository. The suggested model comprises three stages: normalization of data, extraction of weighted normalized features, and prediction. The researchers focus on using optimal features through a hybrid DL approach, specifically recurrent neural networks and Deep Belief Networks (DBN), to make predictions. Anil Kumar Dubey^21^ proposed a DL approach, L-BOA-NN + DBN, for identifying multiple diseases. To evaluate the proposed approach, the author collected datasets from Kaggle and UCI repositories, containing several diseases. The experimental outcomes demonstrated that the proposed design attained favorable results.

Beyond this, Table 1 represents the comparative analysis of a MltD diagnosis system and includes a few studies on single disease diagnosis using DL models. In MltD diagnosis system, researchers used the dataset of various diseases and mostly applied an ML classifier for the classification. In the case of single disease diagnosis, ensemble and DL approaches demonstrate strong performance within individual modalities, and remain limited in their ability to jointly learn from heterogeneous medical data.

**Table 1:** Comparative analysis of methodologies and technologies for multiple disease prediction across different datasets.

Paper	Methodology	Dataset used	Key Findings	Future Directions
^22^	ML and DL approaches	Dataset of various diseases from sources like NHANES, UCI repositories	A comparative study of algorithm performance measures was conducted.	Incorporation of the Internet of Things (IoT). Investigation of multiple feature inputs and hybrid models.
^23^	Strassen’s Rectilinear Fine-tune Bouncing Training (SRFBT) algorithm	Various UCI ML healthcare datasets were used.	The average training time can be up to 43.6249, and the maximum accuracy is equal to 96.86 for the SRFBT algorithm.	-
^24^	ML classification algorithms	Cardiovascular, heart, stroke, Alzheimer’s, breast, and lung cancer dataset.	The voting Classifier performed best in predicting most diseases. However, SVM and AdaBoost also obtained high accuracy for disease prediction.	Hybrid and other DL approaches can be used to achieve good performance.
^25^	Convolutional Neural Networks (CNN) based integrated model	Combination of BRATS 2015 and ISLES 2015	Average accuracy: 99.56% Specificity: 99.99%, Precision: 99.59%, F1-score: 99.57%	Exploring the use of different medical imaging modalities for detection.
^26^	DenseNet121 as the backbone and Chex NeXT	X-ray images of COVID-19, pneumonia, and tuberculosis.	High accuracy results with an average AUC being 0.935.	Future studies will focus on improving metrics for diseases underrepresented through better kinds of images.
^27^	Supervised ML	55,680 patient records	The decision Tree algorithm achieved 94% accuracy in predictions.	Managing large volumes of hospital server data. Enhancing accuracy in remote patient disease prediction.
^28^	Weighted CNN with dilated gated recurrent unit	Cardiovascular, diabetes, pneumonia, and cancer.	Outperforms baseline models (traditional CNN, LSTM, GRU)	Expansion to real-time applications (e.g., IoT-based health monitoring) Integration with Electronic Health Record (EHR) for personalized predictions.
^29^	An ensemble learning approach combining multiple DL models	Cardiovascular diseases, diabetes, cancer, pneumonia, and COVID-19 dataset.	Improved generalization across different disease categories.	Deployment in real-world clinical settings and mobile health applications. Exploring federated learning for privacy-preserving disease diagnosis.
^30^	Deep Neural Networks for Accuracy Enhancement. Broad Learning combined with a denoising autoencoder.	Hybrid Deep Neural Network	Achieved an accuracy of up to 98.50% in disease prediction.	In Real-world deployment and Multi-modal Learning.
^31^	A DL-based multimodal data fusion framework proposed	EHR and medical imaging of various diseases.	Accuracy of up to 98.50%	Challenges in multisource information processing systems have been identified.
^32^	SVM	Diabetes, heart disease, kidney disease, Parkinson’s, and breast cancer.	Accuracy is 95% with SVM	Include additional diseases for a comprehensive prediction system. Explore more ML algorithms to improve accuracy.
^33^	Long Short-Term Memory	A large-scale EHR dataset	The proposed model achieved an F1 score of 88.0%, outperforming several traditional ML and DL models.	Expanding the target output space to include more diseases, incorporating additional demographic and clinical features to improve prediction accuracy
^34^	Bee-Inspired CNN for MltD classification	UCI dataset of Heart, Kidney, and Liver Disease.	Achieved superior accuracy compared to traditional CNN and other DL models.	Exploring hybrid architectures combining BFCNN with transformers or attention mechanisms. - Expanding datasets to include real-world multi-modal medical data.
^35^	Ensemble Learning	UCI and Kaggle datasets of various diseases	Achieved 93.80% Accuracy	DL Integration and use of the Image dataset.

From the literature survey and the summarized table, it has been concluded that most studies suffered from the below-listed challenges in the case of MltD diagnosis.

1. Predominantly relying on EHR datasets. In addition to this, ML classifiers have been implemented for MltD diagnosis.
2. Faced challenges in handling the diverse modalities of images like X-rays, CT scans, ultrasounds, etc.
3. Dealing with highly imbalanced and insufficient datasets.

The proposed research mainly focuses on addressing these challenges and provides an efficient solution for MltD diagnosis using the image dataset.

## Materials and methods

The trend in the 21 st century sees computer science, the medical sciences, and the healthcare sectors collaborating and resolving various medical-related challenges. All these use advanced technologies such as AI, Cloud Computing, and the IoT to generate huge amounts of medical data in the form of images, videos, text, audio, and signals. Such data can be studied and researched by people on behalf of medical practitioners in order to create a solution based on an autonomously functioning system for early detection of diseases and successful treatment before they have turned into life-threatening conditions. In addition, powerful models and architectures are required to optimize data and develop algorithms that can be generalized for better healthcare purposes. The discussion on the dataset and the method used in this research follows in the sections below.

### Dataset used

**Fig. 1:**
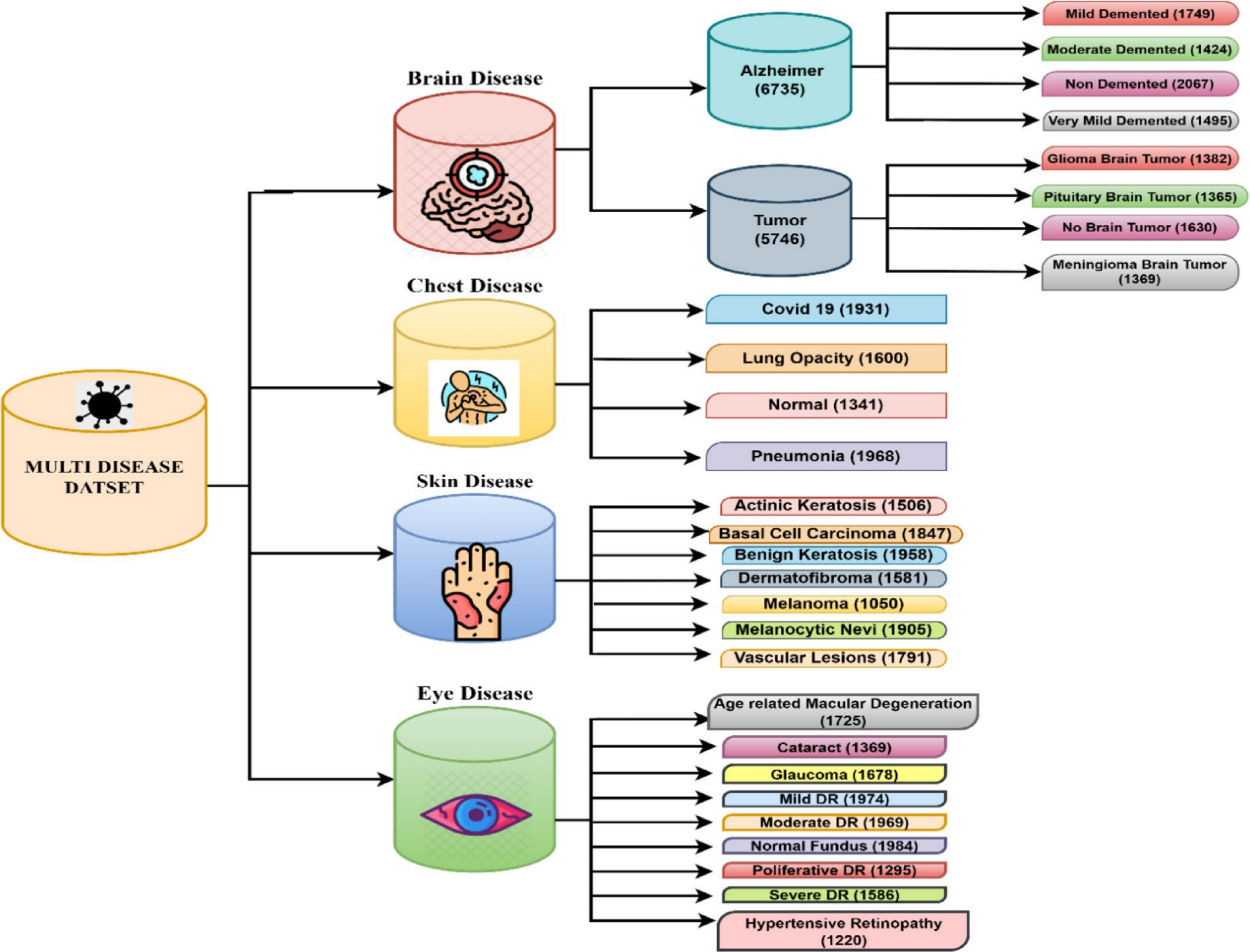
Categorization of Disease.

The dataset used for the proposed work is taken from the open-source Kaggle platform, which encompasses different diseases, categorized into four primary subclasses: brain, chest, skin, and eye disease^36^. Under each subclass, there are innumerable sub-diseases, which form a complicated database of medical disorders. The analysis incorporates diverse sub-diseases within the field of brain disease. To the brain, these sub-diseases manifest as many neurological diseases, such as tumor-associated diseases and cognitive disorders. Chest disease has subelements, such as various respiratory disorders, including lung opacity, pneumonia, etc. The data set utilized in the field of dermatology consists of different types of skin lesions, which are unusual dermatological diseases with health peculiarities and symptoms. Even more than this, the subcategory of eye diseases offers a wide range of ocular diseases, including the dainty complexities of retinopathy and cataracts, or the inexorable progression of glaucoma disease. All these subclasses of the research work data are represented in Fig. 1.

Table 2 offers the dataset characteristics, including the distribution of various classes, sample size, and distinguishing features. This information provides an appropriate understanding regarding the dataset formulation, thus assisting one in understanding the structure and suitability of the subject for analysis.

**Table 2:** Dataset Characteristics.

Category	Details
Total Size of Dataset	45,759 images
Dataset Split	Training: 36,598 (80%), Validation: 4,598 (10%), Testing: 4,563 (10%)
Modalities	X-rays (chest, some brain), CT scans (chest, brain), MRI (brain), Dermoscopic images (skin), and ocular images (eye).
Annotations	Provided by certified radiologists & dermatologists - Encoded as 28-dimensional binary vectors (e.g., [1, 0, …, 1]) representing co-occurring diseases.
Class Imbalance	Rare classes: VASC (< 1%), Common classes: Pneumonia (> 15%), Addressed using weighted loss functions.
Ethical Considerations	Data anonymized per HIPAA/GDPR - Efforts made to balance sampling for fair representation.

**Fig. 2:**
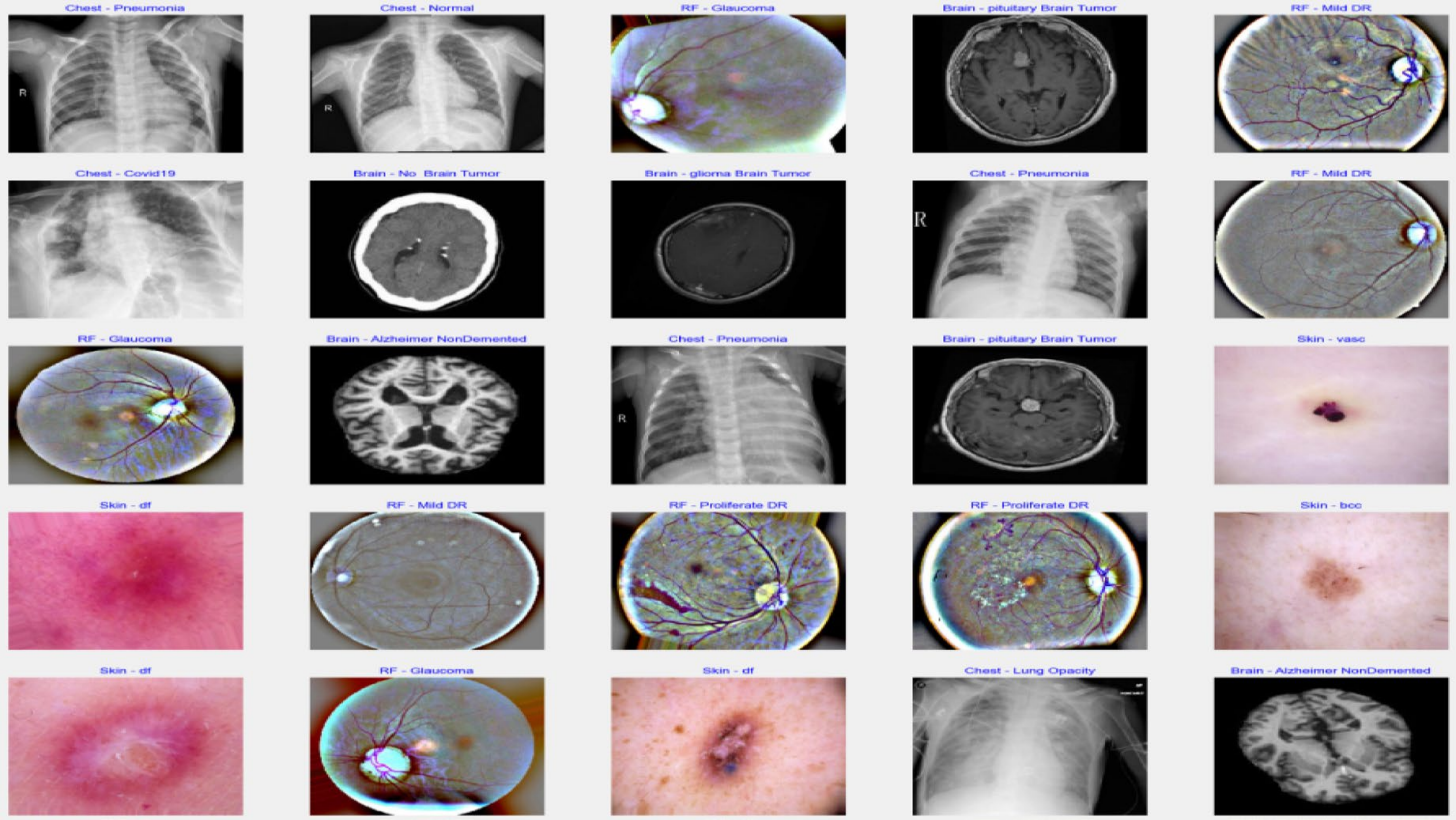
Sample of Disease Images.

The dataset incorporated in the work represents a wide range of more than 45,759 medical images of different modalities that are linked to various diseases, including brain, pulmonary, dermatological, and ocular problems^36^. These massive and diverse data can serve as a strong base on which a strong model can be established that is capable of detecting multiple illnesses using medical imaging data, which would raise the possibility of clinical decision support. Figure 2 displays a sample set of the images of each category.

### Data pre-processing

**Fig. 3:**
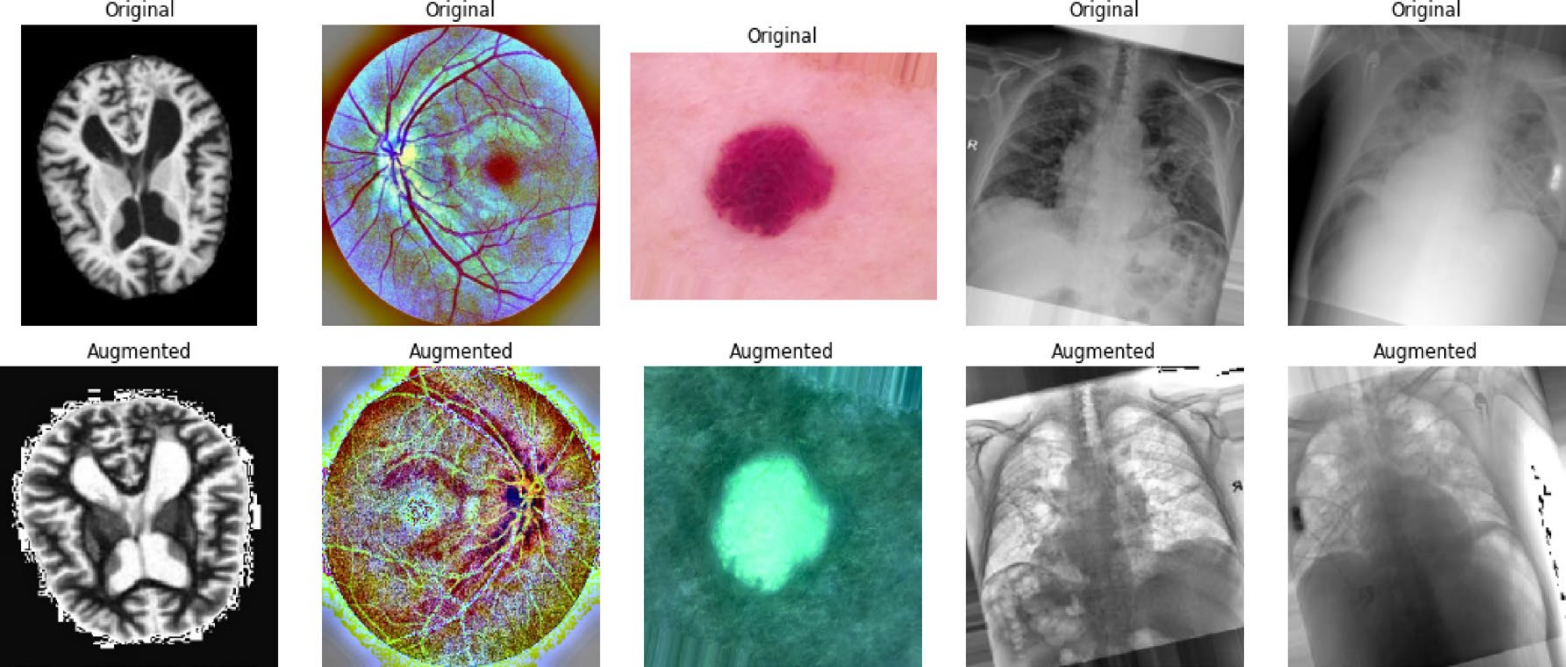
Qualitative Comparison Between Original and Augmented Images.

Preprocessing of data is required to make sure that the model is trained using high-quality data. By utilizing the method of resizing, augmentations, and Fourier transforms, input and feature visibility standardization of reliable model training enhancement is achieved. The sequence of pre-processing steps employed for the work is presented below. Moreover, a sample of some input images and the corresponding results of the data augmentation stage are represented in Fig. 3.

- Load Dataset: Images and annotations are imported from the augmented data directory.
- Resizing: Images are resized to 224 × 224 pixels for all models to maintain consistency across architectures and to ensure computational efficiency.
- Rescaling & Normalization: Pixel values are normalized to [0, 1] via min-max scaling, followed by standardization to ImageNet means (0.485, 0.456, 0.406) and standard deviations (0.229, 0.224, 0.225).
- Enhancement: Contrast Limited Adaptive Histogram Equalization (CLAHE, clip limit = 2.0, tile grid size=8 × 8) improves contrast in X-rays and CT scans.

### Augmentation

- Random Brightness/Contrast (limits = 0.3): Simulates lighting variations.
- Gauss Noise (var_limit = 10–50): Adds robustness to sensor noise.
- Grid Distortion (num_steps = 5, distort_limit = 0.3, *p* = 0.5): Mimics anatomical deformations.
- Elastic Transform (alpha = 1, sigma = 50, alpha_affine = 50, *p* = 0.5): Models tissue elasticity.
- Cutout (1-hole, max size=20 × 20, *p* = 0.5): Encourages focus on non-occluded regions.
- Shift-Scale-Rotate (shift = 0.1, scale = 0.1, rotate = 20°, *p* = 0.5): Enhances spatial invariance.

### Fourier transformations

**Fig. 4:**
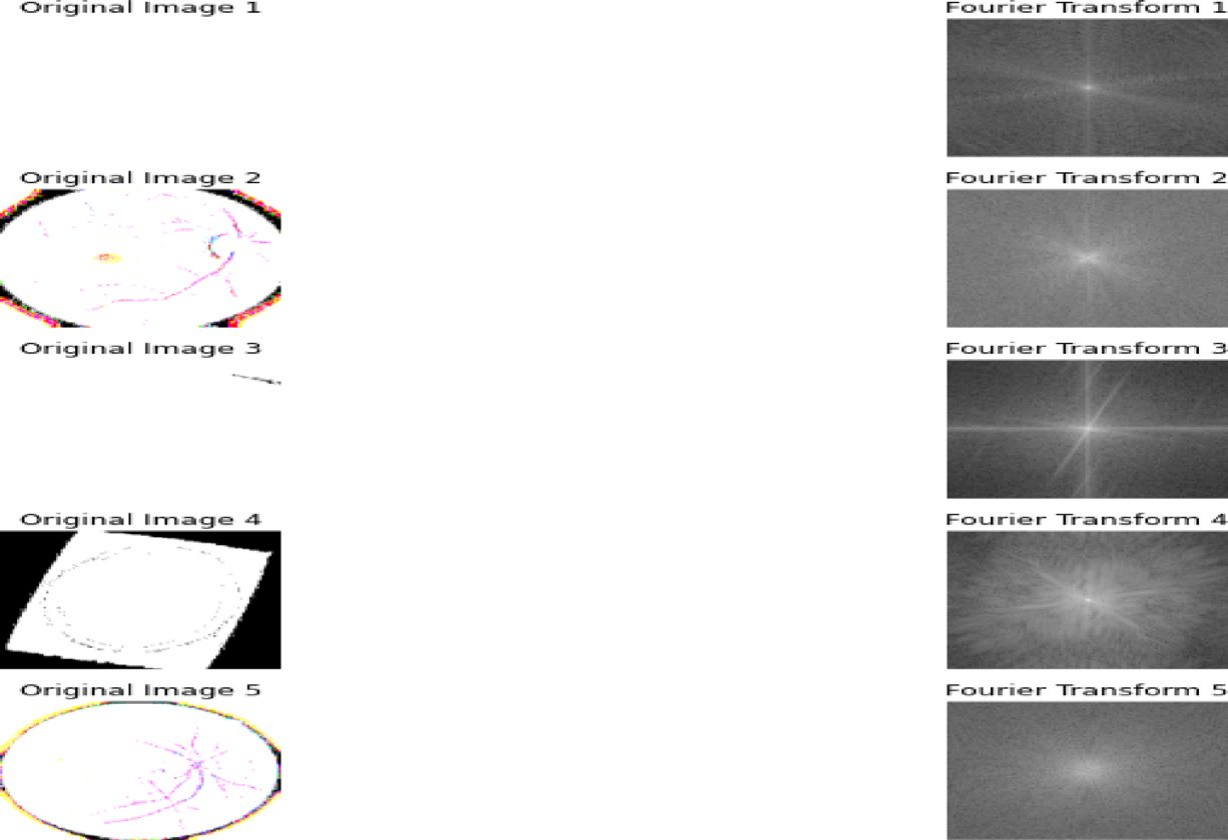
Sample Fourier Transformed Images.

Frequency-domain analysis is applied to select images (e.g., chest X-rays) using Fast Fourier Transform (FFT) to suppress low-frequency noise (e.g., background gradients) while preserving high-frequency disease features (e.g., lung opacities). A high-pass filter with a cut-off frequency of 0.1 cycles/pixel is used, followed by an inverse FFT to reconstruct enhanced images. The output of this is shown in Fig. 4.

### Split dataset

Splitting ensures robust model evaluation and generalization, allowing the model to be trained on a substantial portion of the data while keeping separate sets for hyperparameter tuning and evaluating model performance. Initially, the dataset is split into an 80:10:10 ratio (training, validation & testing ratio).

To ensure the unbiased evaluation of the model, a 5-fold Stratified K-fold cross-validation procedure was also employed for the research work. This strategy splits the dataset into five mutually exclusive subsets, each maintaining the original class distribution to counterbalance the potential effects of class imbalance. For each fold, one subset is tested while the remaining four are used for training. The same process continues until each subset has been used as a test once. Performance is then reported as the mean ± standard deviation of that from all folds.

### Proposed methodology

**Fig. 5:**
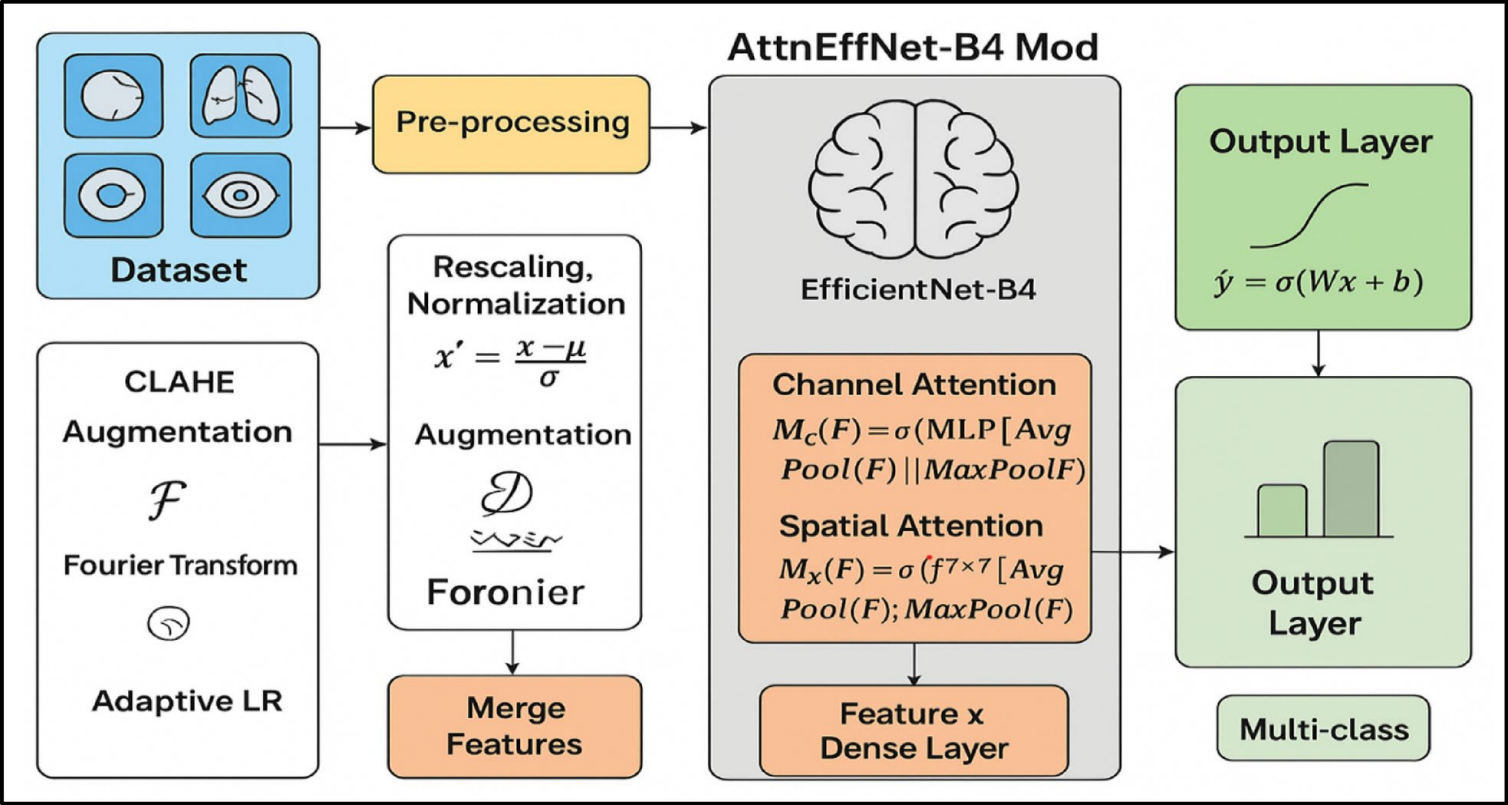
Architectural Diagram of AttnEffNet-B4 Model.

**Fig. 6:**
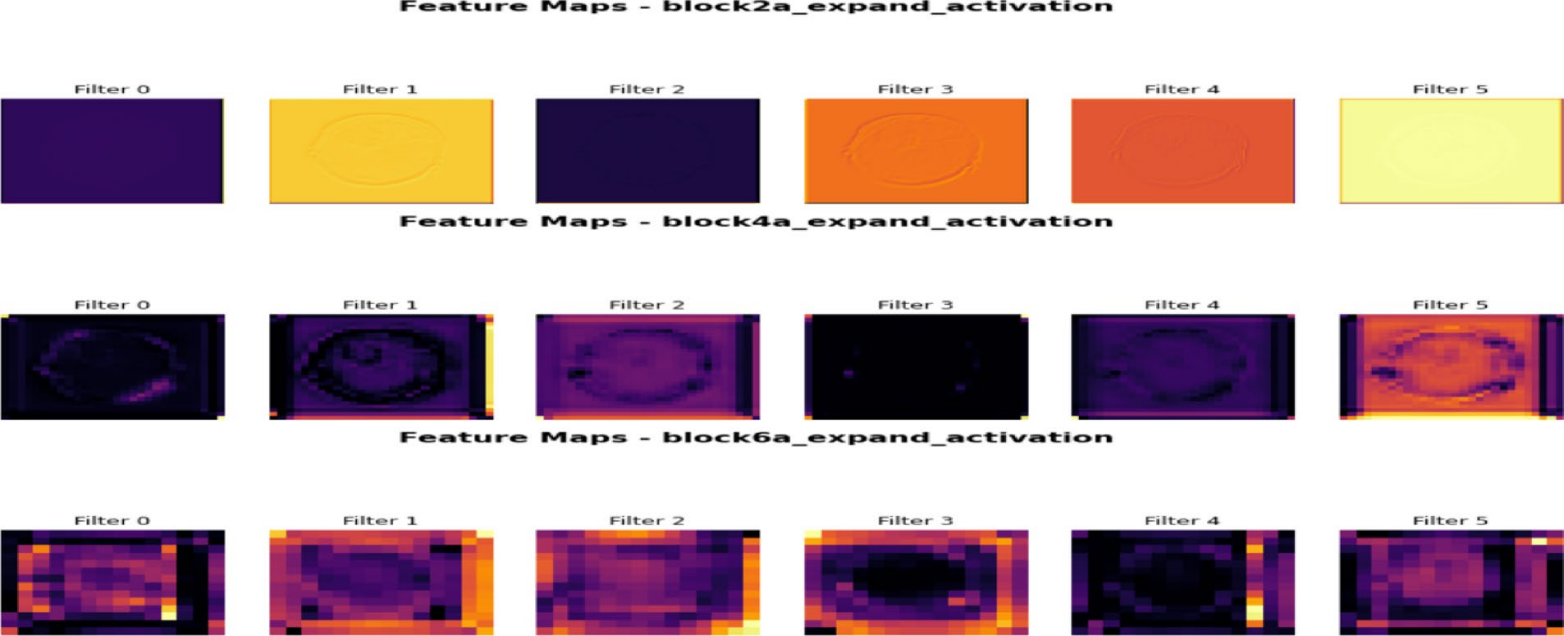
Feature Maps of Intermediate layers.

To address the MltD classification task using image data, the study proposed a novel AttnEffNet-B4 model that takes an input dataset of four different modalities, such as X-rays, CT Scans, skin lesions, and ocular images. Afterwards, the proposed approach leverages advanced pre-processing techniques with Fourier transformation, EfficientNet-B4 as a backbone, and transfer learning with an attention mechanism to improve medical image analysis, as presented in Fig. 5. Prior to feature extraction, the Fourier transformation is employed in order to amplify the features of pathological patterns in the frequency domain. By utilizing pre-trained EfficientNet-B4 weights, originally trained on the ImageNet dataset, the model benefits from existing knowledge, improving its generalization on limited medical image data. Further, the AttnEffNet-B4 model incorporates a self-attention mechanism that applies channel attention to reweight the feature maps and spatial attention to enhance performance by focusing on relevant image features and detecting subtle patterns. AttnEffNet-B4 architecture utilizes attention mechanisms to enable effective feature selection by obtaining attention scores from the dot product of feature representations, followed by the application of the softmax to obtain attention weights. These weights are then used to weight the feature representations, resulting in attended features that are concatenated with the original ones. AttnEffNet-B4 models process data through several levels of abstraction, and visualizing the outputs of intermediate layers provides insight into how a model learns hierarchical features, recognizes patterns, and makes decisions, as presented in Fig. 6. This procedure is particularly valuable in various fields like computer vision, natural language processing, and medical applications, where interpretability is critical.

**Fig. 7:**
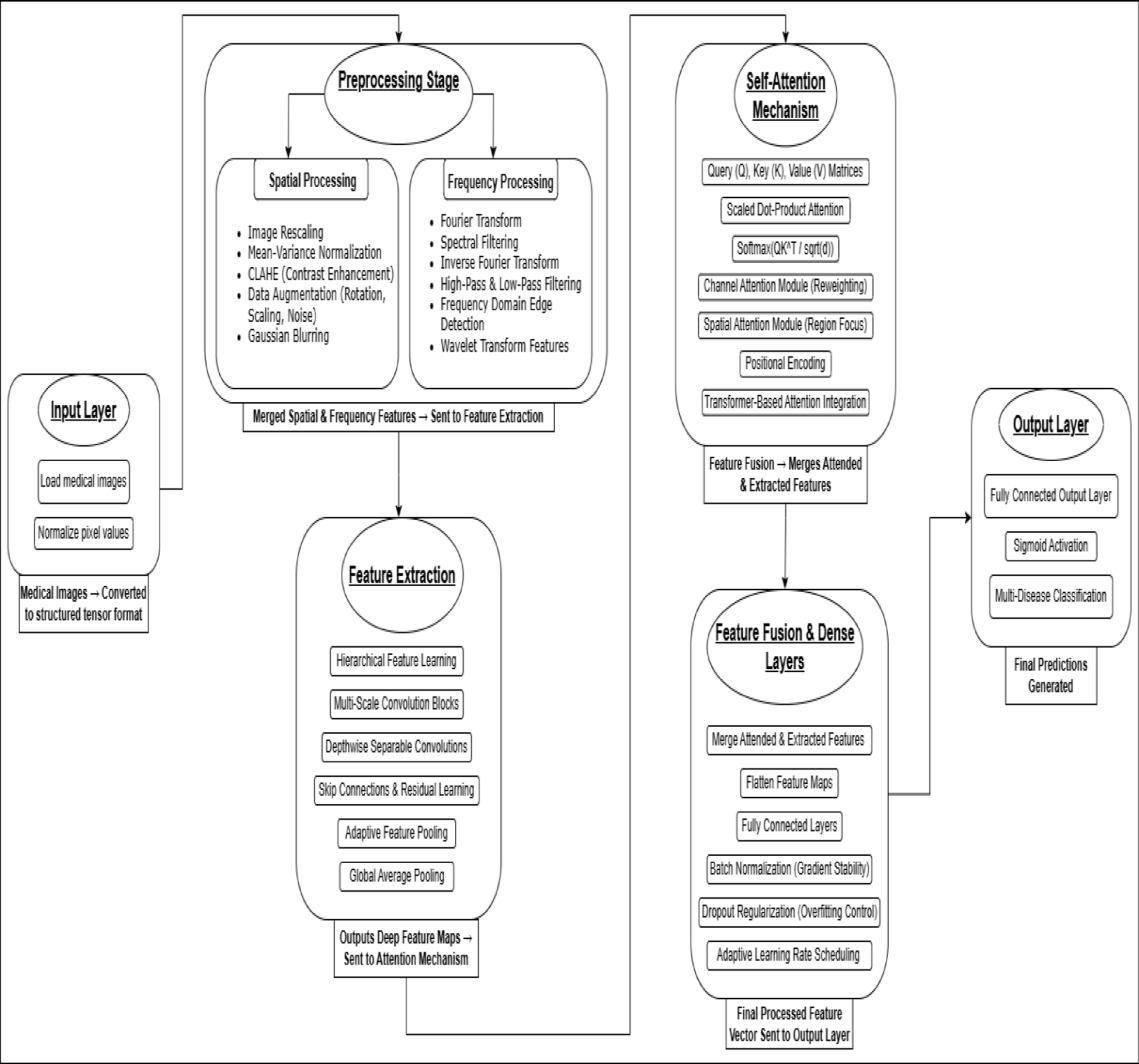
Block Diagram of the Proposed AttnEffNet-B4 model.

The model architecture consists of four primary stages: pre-processing stage & feature extraction, attention mechanism, model building & training, and evaluation as presented in Fig. 7. The methodology aims to design a robust DL framework that can classify different subtypes of diseases. This study is intended to raise the standards of medical diagnostics and advances in healthcare by using advanced DL techniques.

### Algorithm of proposed AttnEffNet-B4 model

#### Step 1: dataset preparation

Let the input image dataset be:

$$\:D=\left\{\left({X}_{i,\:},\:{Y}_{i}\right)\right\}\:\:\forall\:\:i\in\:[1,\:N]\:$$

Where:

- $\:{X}_{i\:}\in\:\:{R}^{H\times\:W\times\:C\:}$where $\:{X}_{i\:}\:$is an input image (height $\:H$, width $\:W$, channels $\:C$) and R is a set of real numbers.
- $\:{Y}_{i\:}\in\:{\{0,\:1\}}^{M}$ is the labeled disease classification vector (M classes).
- $N$ is the total no. of images.

#### Step 2: dataset pre-processing

Various pre-processing techniques have been applied to the input dataset.

i. Resizing.

$${X_i}\left( {224 \times 224} \right)$$

ii. Rescaling.

$$\:{X}_{i}^{{\prime\:}}=\:\frac{{X}_{i,\:}}{255}$$

iii. Normalization and enhancement:

$$\:{X}_{i}^{{\prime\:}{\prime\:}}=\frac{{X}_{i}^{{\prime\:}}-\:\mu\:}{\sigma\:}$$

$\:\mu\:$ = Mean, $\:\sigma\:$ = Standard deviation.

CLAHE (clip limit = 2.0, tile grid size = 8 × 8).

iv. Augmentations (Transform $\:T$):

$$\:{X}_{i}^{{\prime\:}{\prime\:}{\prime\:}}=T\left({X}_{i}^{{\prime\:}{\prime\:}}\right)$$

v. Fourier transform:

$$\:F\left(u,v\right)=\sum\:_{x=0}^{H-1}\sum\:_{y=0}^{W-1}X(x,y){e}^{-j2\pi\:(ux/H\:+\:vy/W)}$$

Where:

- $\:F\left(u,v\right)$ represents the frequency domain transformation at frequency coordinates $\:(u,v$) of the image $\:X\left(x,y\right)$ | (x and y are the coordinates of the image).
- $\:(\frac{ux}{H}and\:\frac{vy}{W}$ are the Normalized spatial frequency terms that relate spatial positions $\:\left(x,y\right)\:$to frequency indices $\:(u,v$).

#### Step 3: AttnEffNet-B4 model construction

The backbone network is EfficientNet-B4, denoted as $\:{f}_{\theta\:}$, where:

$$\:\mathrm{Z}\:=\:{f}_{\theta\:}\left(X\right)\:\mathrm{w}\mathrm{h}\mathrm{e}\mathrm{r}\mathrm{e}\:Z\in\:\:{R}^{h\times\:w\times\:d\:}$$

- Z is the extracted feature map.
- $\:h,\:w$ are spatial dimensions.
- $\:d$ is the number of feature channels.

#### Step 4: self-attention mechanism

i. Compute query (Q), Key (K), and Value (V) metrics:

$$\:Q={W}_{Q}Z,\:K={W}_{K}Z,\:V={W}_{V}Z$$

Where $\:{W}_{Q}$, $\:{W}_{K}$, $\:{W}_{V}$ are learnable weight metrics.

ii. Compute attention scores:

$$\:A=Softmax\left(\:\frac{Q{K}^{T}}{\sqrt{d}}\:\right)$$

Where $\:A\in\:\:{R}^{d\times\:d\:}$is the attention matrix.

iii. Compute attended features:

$$\:{Z}^{{\prime\:}}=AV$$

iv. Residual connection & normalization:

$$\:{Z}_{attended}=LayerNorm({Z}^{{\prime\:}}+Z)$$

#### Step 5: fully connected layers

After attention, feature maps are flattened and passed through a dense layer:

$$\:F=Flatten\left({Z}_{attended}\right)$$

$$\:{F}_{1}=\sigma\:({W}_{1}F+\:{b}_{1})$$

Where:

- $\:{W}_{i}$, $\:{b}_{i}$ are weights and biases of dense layers, and the i value varies between 1 to n.
- $\:\sigma\:\left(x\right)=ReLU\left(x\right)$ is the activation function.

#### Step 6: output layer

Since this is label classification, the final output uses the sigmoid function for each class.

$$\:\widehat{Y}=\:\sigma\:({W}_{o}{F}_{1}+\:{b}_{o})$$

Where $\:\widehat{Y}\in\:{[0,\:1]}^{M}\:\:$is the predicted probability for each of the $\:M$ disease labels.

#### Step 7: loss function

Binary cross-entropy (BCE) is used for multi-label classification:

$$\:\mathcal{L}=-\frac{1}{N}\sum\:_{i=1}^{N}\sum\:_{j=1}^{M}[{Y}_{ij}\mathrm{log}\left({\widehat{Y}}_{ij}\right)+(1-{Y}_{ij})\mathrm{l}\mathrm{o}\mathrm{g}(1-{\widehat{Y}}_{ij})]$$

Where:

- $\:{Y}_{ij}$is the true label.
- $\:{\widehat{Y}}_{ij}$ is the predicted probability.

#### Step 8: model training

To optimize the model, we use the Adam optimizer with learning rate scheduling:

$$\:{\theta\:}_{t+1}={\theta\:}_{t}-\eta\:.\frac{{m}_{t}}{\sqrt{{v}_{t}}+\epsilon}$$

Where:

- $\:{\theta\:}_{t\:}$= learnable parameters at the current training step (before update).
- $\:{\theta\:}_{t+1}$= learnable parameters after update.
- $\:{m}_{t}$ = first moment estimate and $\:{v}_{t}$ = second-moment estimate.
- $\:\eta\:$ = learning rate (adjusted dynamically) and $\epsilon$ = small constant to avoid division by zero.

Batch training: For each batch –

- Forward Pass: Compute predictions $\:Y$.
- Compute Loss: Compute $\:\mathcal{L}$.
- Backward Pass: Compute gradients $\:{\nabla\:}_{\theta\:}\:\mathcal{L}$.
- Update Weights: Using Adam optimizer.

#### Step 9: evaluation metrics

After training, evaluate using:

i. Accuracy:

$$\:Accuracy=\frac{\sum\:_{i=1}^{N}1({\widehat{Y}}_{i}={Y}_{i})}{N}$$

ii. Precision, recall, and F1-score:

$$\:Precision=\frac{TP}{TP+FP}$$

$$\:Recall=\frac{TP}{TP+FN}$$

$$F1 - Score = \frac{{2 \times \Pr ecision \times \operatorname{Re} call}}{{\Pr ecision + \operatorname{Re} call}}$$

Where:

- TP: True Positives(Number of correctly classified positive samples).
- FP: False Positives(Number of negative samples incorrectly classified as positive).
- FN: False Negatives(Number of positive samples incorrectly classified as negative).

iii. Confusion matrix $\:C$:

$\:{C}_{ij}$ = Count of samples with true label i predicted as j.

### Computational complexity

The AttnEffNet-B4 model that is proposed has its computational complexity primarily determined by the backbone of EfficientNet-B4 and the fully connected dense layer. The EfficientNet-B4 backbone has about 17.7 million parameters and prevails in the convolutional feature extraction process. After feature extraction, the feature maps are converted into a one-dimensional array of 87,808 features in the form of a Flatten operation, and then the Flatten operation is followed by a Dense layer. The suggested design includes a compact dense block that is composed of a 64-unit fully connected layer that adds about 5.6 million parameters, followed by the addition of batch normalization and a final classification layer. Despite the fact that the inclusion of the flatten operation and dense block causes the total number of parameters to nearly 23.3 million, the model still successfully maintains its discriminative performance. In addition to it, the attention mechanism slightly adds to the computational complexity with its channel-wise and spatial weighting operations, while the Fourier transformation is done through the FFT. Theoretically, the model can be approximated to have a computational complexity of

$$\:O\left(N\cdot\:C\cdot\:H\cdot\:W\right)+O\left(NlogN\right)+O\left(N\cdot\:M\right)$$

The first term is convolutional feature extraction, the second is the Fourier transformation, and the third is the fully connected layers, where N denotes the number of input features, M represents the output dimensions of the fully connected layer, and C, H, and W denote the feature map channel, height, and width, respectively. Despite the increment of the number of parameters, the Dense layers allow more interactions between the features and more discriminative learning in a heterogeneous set of diseases. So, even though the extra components are added, the model still offers a good trade-off in terms of computational efficiency and diagnostic accuracy, and remains feasible in modern real-time healthcare settings.

## Results and discussion

The main findings of the suggested methodology are discussed in this section. The performance evaluation begins with the state-of-the-art models, followed by the comparative analysis with the proposed AttnEffNet-B4 model. Various models, such as InceptionV3, EfficientNetB0, EfficientNetB4, ResNet50, VGG16, and VGG19, have been tested on medical image datasets covering brain diseases (Alzheimer’s, Brain Tumors), chest conditions (COVID-19, Lung Opacity, Pneumonia), retinal fundus (RF), and skin lesions. The performance of all the models was evaluated using a hybrid computational system based on a local workstation and a cloud-based demonstration of the GPU environment to ensure flexibility and computational efficiency. All development, data preprocessing, and initial experimentation were conducted using a local machine with an Intel Core i5-11300 H processor with a frequency of 3.10 GHz, 16GB RAM, and Windows 11 Home operating system, which is a 64-bit operating system. To achieve computationally intensive computations, including model training and fine-tuning, a GPU-enabled environment was used by using Google Colab, where the NVIDIA T4 GPU was used to speed up the deep learning work. The execution of the proposed model was conducted on the basis of the Keras deep learning framework, which allowed for efficient model design, training, and evaluation. The parameters employed for the proposed model training are presented in Table 3. This hybrid implementation plan allowed the ideal use of opportunities available through optimization of local processing performance and acceleration in the cloud, and thus enhanced the efficiency of training and minimized the overall time spent on computations. Additionally, accuracy and loss graphs of training and validation performance over epochs are illustrated and evaluated, showing the model’s ability to learn disease classification. The loss graph highlights the model’s error reduction and indicates its convergence toward accurate predictions.

**Table 3:** Parameters for the proposed model training.

Hyperparameter	Value	Hyperparameter	Value
Image Size	224 × 224 × 3	Activation Function	ReLU
Number of Classes	28	Output Activation	Softmax
Train/Validation/Test	80%/10%/10%	Optimizer Type	Adam
Batch size (Train/Test)	25/32	Learning Rate	0.001
Epochs	15	Momentum	0.9
Pre-trained Model	EfficientNet-B4	Loss Function	Categorical Cross-Entropy
Pre-training Dataset	ImageNet	Augmentation Techniques	CLAHE, Rotation, Gauss Noise, Cutout, Grid Distortion
Dense layer	64		

### Performance analysis of the traditional CNN and proposed AttnEffNet-B4 model

The section further deals with the impact of each model and its features. Table 4 shows the validation and training accuracy and loss obtained for the VGG-16 Model. VGG-16, developed by the Visual Geometry Group at Oxford, is a deep CNN with 16 layers that includes 13 convolutional layers with 3 × 3 filters, 5 max-pooling layers (2 × 2 stride), and 3 fully connected layers. Therefore, the consistent and deep architecture allows for strong feature extraction^37^. The training with this model has been carried out over 15 epochs for a learning rate of 0.001. The model has achieved a training accuracy of 65.54%. According to Table 4, the training accuracy of this model increases continuously while the validation loss does not decrease gradually concerning the initial epoch of the model, which is not preferred. The model tested on the test dataset produced 71.43% accuracy.

**Table 4:** VGG-16 model performance.

S. No.	Model	Epochs	Learning Rate	Training Accuracy	Training Loss	Validation Accuracy	Validation Loss
1	VGG-16	1	0.001	0.2144	6.1905	0.3943	2.6502
2		4		0.4969	2.0929	0.5694	1.4545
3		8		0.594	1.2844	0.643	1.0475
4		12		0.6385	0.9942	0.6651	0.8816
5		15		0.6554	0.9029	0.6768	0.8326

VGG-19 is a deep CNN comprised of 19 layers, which are specifically designed for image classification tasks. It consists of 16 convolutional layers with small 3 × 3 filters for precise feature extraction, 5 max pooling, 3 fully connected layers, and a SoftMax output layer. It also uses a technique called Max Pooling, which is used to reduce the size of the image. It has a simple and highly accurate architecture along with transfer learning capabilities. On an overall basis, VGG-19 performed the same way as VGG-16 with a training accuracy of 65.09%. This model, like VGG-16, was trained for 15 epochs with a learning rate of 0.001. The summary of the results of the VGG-19 system is illustrated in Table 5. The accuracy obtained was 68.42% when the test data was applied to the model.

**Table 5:** VGG-19 model performance.

S. No.	Model	Epochs	Learning Rate	Training Accuracy	Training Loss	Validation Accuracy	Validation Loss
1	VGG-19	1	0.001	0.2018	6.3971	0.3995	2.6589
2		4		0.4969	2.0086	0.5667	1.4477
3		8		0.5863	1.2871	0.6321	1.0423
4		12		0.627	1.0121	0.6736	0.8768
5		15		0.6509	0.9114	0.6804	0.8408

Inception-V3 and ResNet-50 are deep but efficient architectures of CNN with improved utility in image recognition tasks. The major innovations using it for computational efficiency and accuracy include: factorized convolutions, auxiliary classifiers, complex asymmetric convolutions, etc. These CNN models’ superlative and efficient extraction of complex features impacts diagnosis and analysis. The notation on how the Inception-V3 and ResNet-50 models perform is shown in Tables 6 and 7 respectively. Both were trained, like other models, at a learning rate of 0.001 for 15 epochs. The epoch-wise performance of the Inception-V3 model is presented in Table 6, and it gives only 48.72% training accuracy, and on the trial with a test data set, it achieved a test accuracy of 52.46%. Despite its architectural depth and capability to extract multi-scale features, Inception-V3 and almost all other traditional deep networks face various challenges, such as the vanishing gradients problem, due to which proper model training is not feasible. ResNet-50 model tries to resolve such issues using residual connections, which facilitate the model’s ability to omit certain layers, hence streamlining the training process and improving overall accuracy. Despite having 50 deep layers, ResNet50 is still more efficient than its predecessors because of its unique bottleneck architecture that lowers the number of parameters needed while still performing optimally. The training accuracy and the test result achieved on this model are 73.89% and 73.66%, respectively, which is far better compared to the other model results discussed so far.

**Table 6:** Inception-V3 model performance.

S. No.	Model	Epochs	Learning Rate	Training Accuracy	Training Loss	Validation Accuracy	Validation Loss
1	Inception-V3	1	0.001	0.2403	22.7957	0.4014	7.7815
2		4		0.3919	10.7287	0.486	4.9211
3		8		0.4405	6.2927	0.5121	3.6373
4		12		0.4739	4.405	0.5394	2.6891
5		15		0.4872	3.6618	0.5342	2.4027

**Table 7:** ResNet-50 Model Performance.

S. No.	Model	Epochs	Learning Rate	Training Accuracy	Training Loss	Validation Accuracy	Validation Loss
1	ResNet-50	1	0.001	0.4249	1.6368	0.5901	1.0483
2		4		0.6559	0.8711	0.6676	0.8131
3		8		0.7056	0.7379	0.6992	0.743
4		12		0.7272	0.6796	0.7175	0.6965
5		15		0.7389	0.6538	0.7205	0.6868

The EfficientNet-B0 is the fundamental model of Google and is designed for image classification tasks. It uses the concept of compound scaling, which preferably adjusts the depth, width, and resolution to improve the performance of the model with minimal resources. The EfficientNet-B0 models have completely outperformed the former models in terms of performance. The epoch-wise performance of both EfficientNet-B0 and EfficientNet-B4 models is represented in Tables 8 and 9. When tested on the test dataset, both models had the highest precision of 75%, whereas EfficientNet-B0 gave a test accuracy of 73.21%, and EfficientNet-B4 gave a test accuracy of 70.54%.

**Table 8:** EfficientNet-B0 Model Performance.

S. No.	Model	Epochs	Learning Rate	Training Accuracy	Training Loss	Validation Accuracy	Validation Loss
1	EfficientNet-B0	1	0.001	0.4147	1.8017	0.5552	1.2711
2		4		0.6404	0.9625	0.6493	0.9223
3		8		0.6827	0.8368	0.682	0.8266
4		12		0.7015	0.779	0.7028	0.7752
5		15		0.7109	0.7506	0.7164	0.7521

**Table 9:** EfficientNet-B4 Model Performance.

S. No.	Model	Epochs	Learning Rate	Training Accuracy	Training Loss	Validation Accuracy	Validation Loss
1	EfficientNet-B4	1	0.001	0.4213	1.8325	0.5391	1.3883
2		4		0.6181	1.0263	0.622	1.0262
3		8		0.6588	0.8988	0.6564	0.9156
4		12		0.6782	0.8449	0.667	0.8681
5		15		0.6895	0.8164	0.6823	0.8346

Even though EfficientNet-B0 performed a little better when used alone, EfficientNet-B4 was chosen as the backbone because of its enhanced ability to extract features. EfficientNet-B4 is broader and more profound to allow more discriminative and multi-scale representations, which is especially relevant in medical image analysis since subtle texture and structural changes are present. The nature of learned representations is a more important matter than individual accuracy since the backbone is utilized as a feature extractor, and not as a final classifier. Although the model’s performance is also slightly lower compared to the ResNet-50 model, still EfficientNet-B4 is chosen as a base model for the proposed work due to its cost-effectiveness and lightweight nature.

**Fig. 8:**
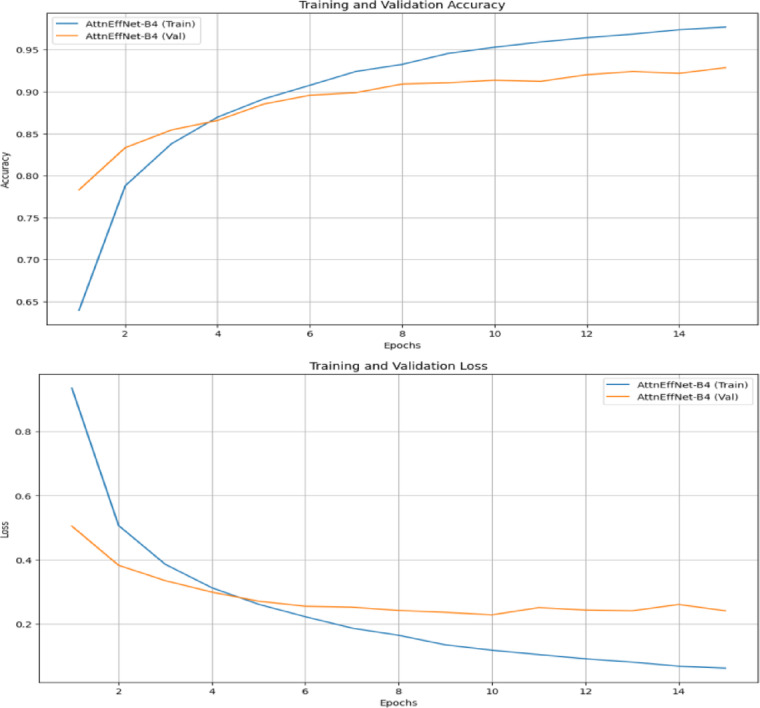
AttnEffNet-B4 Model Accuracy and Loss Graph.

AttnEffNet-B4 is a suggested model that combines advanced DL techniques, including transfer learning and attention. The model was trained on 15 epochs by an Adam optimizer with a learning rate of 0.001 and a batch size of 25, using which the model was fine-tuned to accomplish the best results. AttnEffNet-B4 was constructed as a variant of EfficientNet-B4 with extra attention layers and was created to boost feature extraction, which will result in its superior ability to fulfill its medical image detection tasks with subtle patterns. The proposed model result outperformed the existing pre-trained convolutional network models in terms of accuracy, loss, and efficiency, and also achieved higher performance on a benchmark dataset while reducing computational complexity as presented in Table 10. Moreover, epoch-wise model performance is illustrated in Fig. 8, where the curves phenomenologically reflect the right learning of the model with increasing epochs.

**Table 10:** AttnEffNet-B4 Model Performance.

S. No.	Model	Epochs	Learning Rate	Training Accuracy	Training Loss	Validation Accuracy	Validation Loss
1	AttnEffNet-B4 Model (Proposed)	1	0.001	0.6395	0.9358	0.783	0.5049
2		4		0.8696	0.3123	0.8657	0.2988
3		8		0.9325	0.164	0.9091	0.2416
4		12		0.9643	0.0907	0.9202	0.2429
5		15		0.9769	0.0616	0.9286	0.2405

**Fig. 9:**
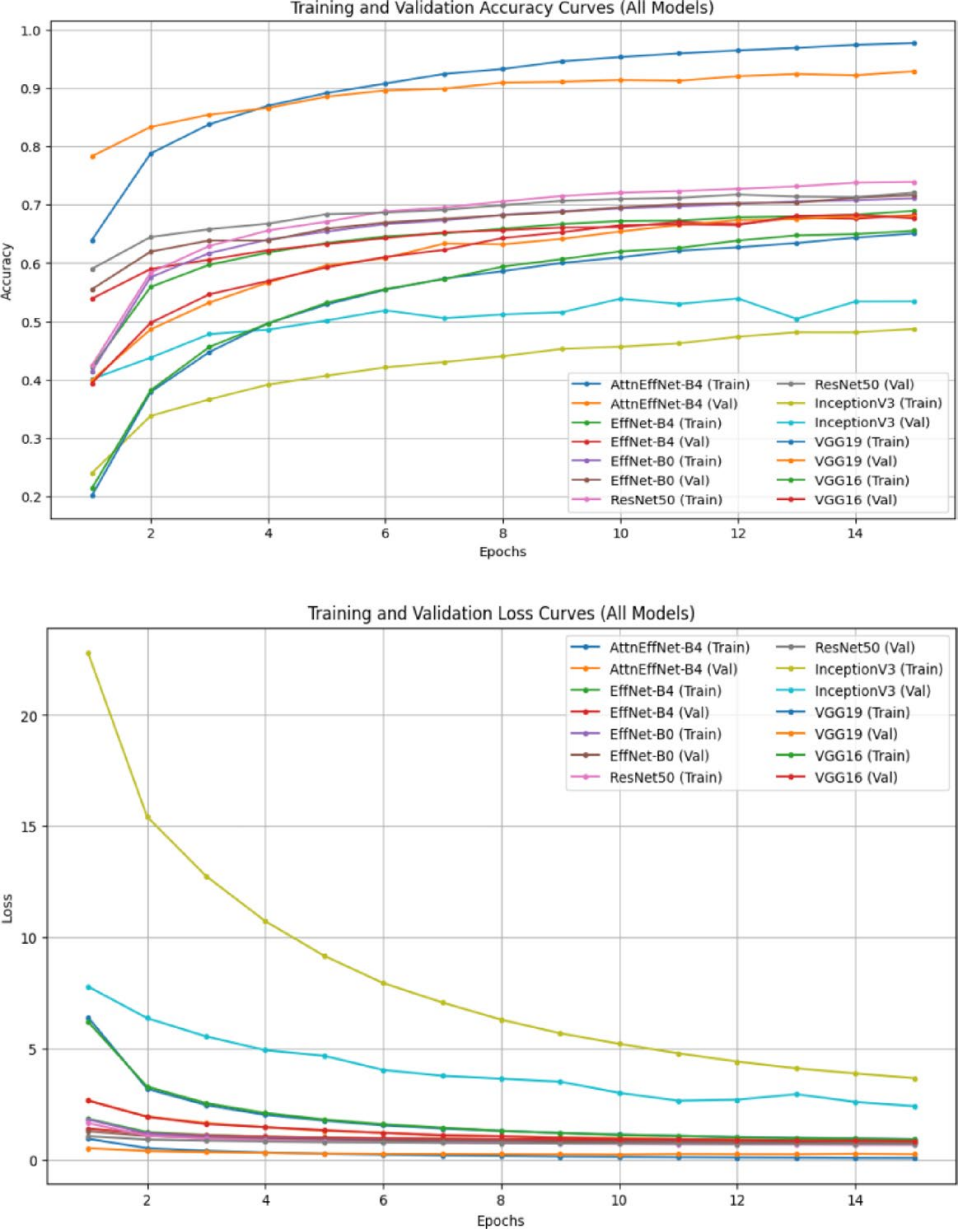
Convergence Analysis of Training and Validation Accuracy and Loss for All Models.

The combined training and validation accuracy and loss curves of all the considered models are shown in Fig. 9, which is a complete comparison of convergence behavior and generalization performance of all models with the number of epochs. In comparison, VGG-16, VGG-19, and Inception-V3 were found to be slower converging, experience higher loss values, and have worse overall accuracy. So, they are less efficient in terms of optimization, and their generalization is poor. Conversely, EfficientNet-B0 and B4 models show more fluid learning curves that have more stable accuracy gains and less loss. Interestingly, the suggested AttnEffNet-B4 has the highest training and validation accuracy and the smallest validation loss, and the least difference between the training and validation curves. This is an indication of a quicker convergence, better stability of learning, and better generalization ability. Coupling the attention processes and dense layers to the EfficientNet-B4 backbone proves to be successful in refining the discriminative features representation, resulting in powerful and stable multi-disease classification rates.

In order to have a thorough evaluation of the computational properties of the proposed model, a comparison was made with other DL models. Unlike lightweight models like VGG-16, VGG-19, InceptionV3, and EfficientNet-B0, the focus of this study is to compare them with a relatively high-capacity model to provide a fair comparison between them, considering similar representational ability. This is compared in critical metrics such as the number of parameters, the cost of computation, training time, the resilience of inference, and the consumption of memory, as tabulated in Table 11.

**Table 11:** Computational and Runtime Performance Comparison.

Model	Parameters (Million)	FLOPs (GFLOPs)	Training Time (Hrs)	Inference Latency (ms per image)	Memory Usage (GB)
ResNet50	25.6	4.1–4.4	2.7–3.4	15–22	3.5–4.4
EfficientNet-B4	17.7	4.2–4.4	2.6–3.3	17–23	3.3–4.1
AttnEffNet-B4 (Proposed)	23.3	4.3–4.8	2.9–3.5	18–24	3.4–4.3

**Fig. 10:**
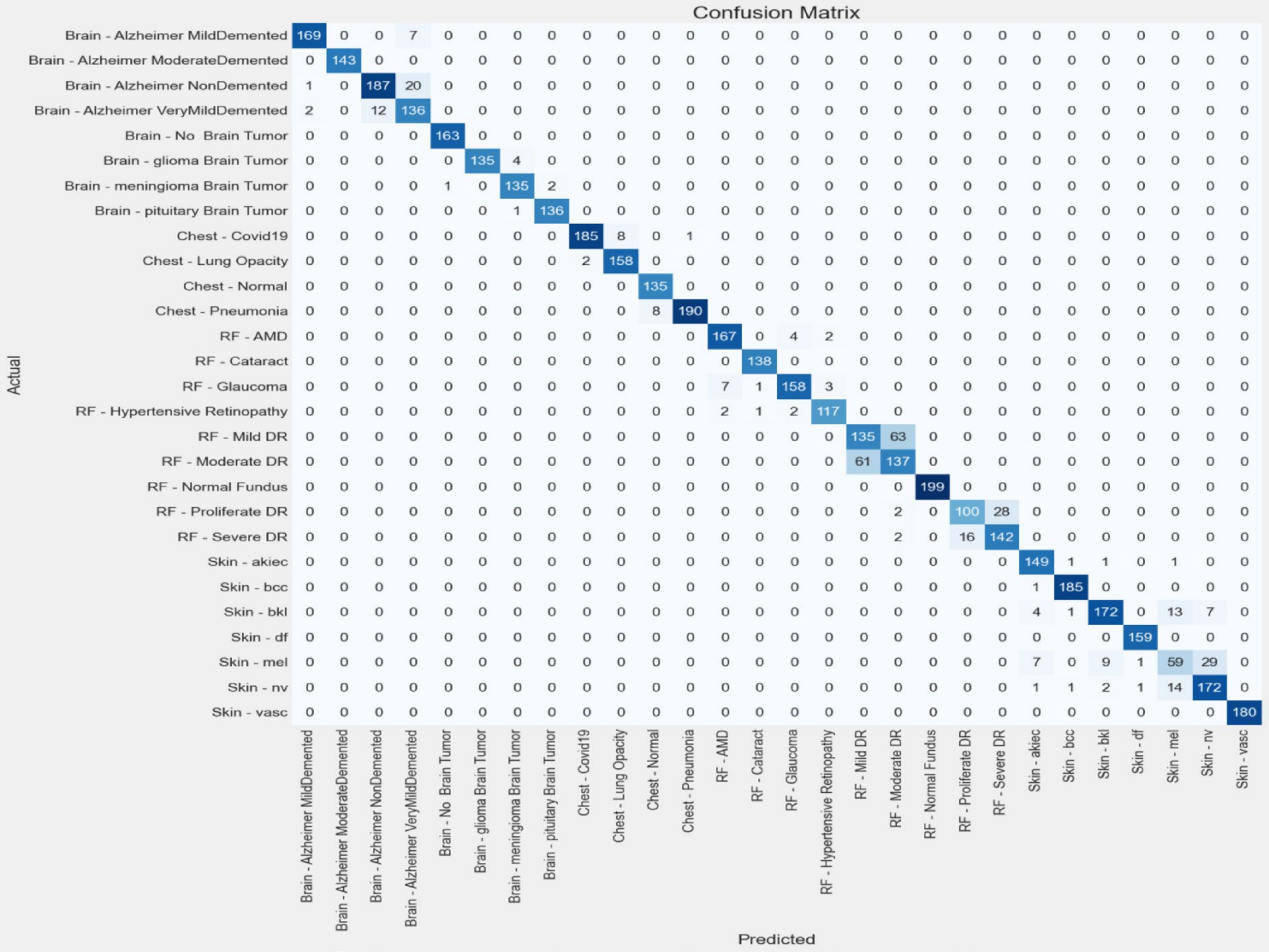
Confusion Matrix for AttnEffNet-B4 Model.

The confusion matrix represents the classification results across brain, chest, retinal fundus, and skin diseases, where each row represents actual classes, and each column represents predicted classes, as shown in Fig. 10.

**Fig. 11:**
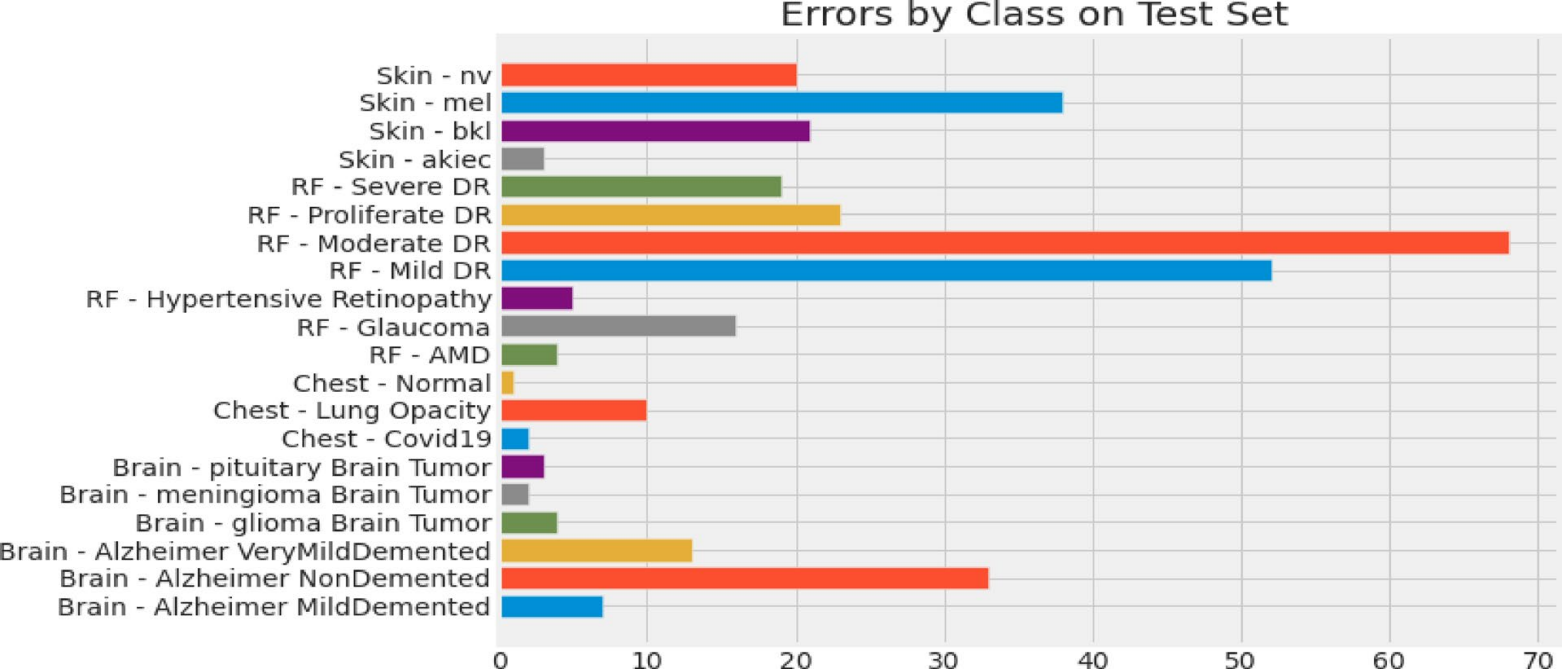
Errors by Class on Test Set for AttnEffNet-B4 Model.

The diagonal of the matrix reflects the right classification, and the terms on off-diagonal reflect the wrong classification. Nonetheless, as the model was able to achieve high performance, it exhibited weaknesses in learning the specific classes, like RF-Moderate DR, RF-Mild DR, because of the retinal image feature overlap, as shown in Fig. 11. These results indicate that the model can be effective in dealing with the MltD disease classifications, but it needs further improvement. Moreover, the classification report shows important figures, such as precision, recall, F1-score, and support on each disease class, which demonstrates the performance of AttnEffNet-B4, as shown in Table 12.

**Table 12:** Performance Evaluation of AttnEffNet-B4 Model on Different Classes.

Class Name	Precision	Recall	F1-Score	Support
Brain - Alzheimer MildDemented	0.98	0.96	0.97	176
Brain - Alzheimer ModerateDemented	1	1	1	143
Brain - Alzheimer NonDemented	0.94	0.9	0.92	208
Brain - Alzheimer VeryMildDemented	0.83	0.91	0.87	150
Brain - No Brain Tumor	0.99	1	1	163
Brain - Glioma Brain Tumor	1	0.97	0.99	139
Brain - Meningioma Brain Tumor	0.96	0.98	0.97	138
Brain - Pituitary Brain Tumor	0.99	0.99	0.99	137
Chest - Covid19	0.99	0.95	0.97	194
Chest - Lung Opacity	0.95	0.99	0.97	160
Chest – Normal	0.94	1	0.97	135
Chest – Pneumonia	0.99	0.96	0.98	198
RF – AMD	0.95	0.97	0.96	173
RF – Cataract	0.99	1	0.99	138
RF – Glaucoma	0.96	0.93	0.95	169
RF - Hypertensive Retinopathy	0.96	0.96	0.96	122
RF - Mild DR	0.69	0.68	0.69	198
RF - Moderate DR	0.67	0.69	0.68	198
RF - Normal Fundus	1	1	1	199
RF - Proliferate DR	0.86	0.77	0.81	130
RF - Severe DR	0.84	0.89	0.86	160
Skin – Akiec	0.92	0.98	0.95	152
Skin – BCC	0.98	0.99	0.99	186
Skin – BKL	0.93	0.87	0.9	197
Skin – DF	0.99	1	0.99	159
Skin – MEL	0.68	0.56	0.61	105
Skin – NV	0.83	0.9	0.86	191
Skin – VASC	1	1	1	180

### Comparative analysis and ablation study

A comparative analysis was also made with various CNN-based models based on conventional measures of performance, and the characteristics of the model comparison are presented in Table 12. Conventional CNN-based models are quite moderate in performance, while more parameter-efficient and deeper networks such as EfficientNet-B0, EfficientNet-B4, and ResNet-50 have managed to achieve a little higher. Comparatively, the AttnEffNet-B4 model proposed had a testing accuracy of 94.47%, a precision of 93%, a recall, and an F1-score of 92%, by far surpassing all the other methods presented as baselines. This huge increase is a sign of how the self-attention mechanism and Fourier transformation offer an advantage where it counts, as it allows the model to capture subtle textural and structural variations in images of diseases.

An ablation study was also conducted, where some modules were added in order to see the overall effectiveness. This was made possible by the selective addition of certain modules in the baseline EfficientNet-B4, which enabled us to measure the contribution made by the individual components towards the overall performance of the model. This discussion was very informative on what components of the network were critical in achieving good feature extraction and correct classification. By integrating EfficientNet-B4 with self-attention, some preprocessing techniques, and dense layers, the model performance is significantly boosted and demonstrates the highest accuracy with the most stable curves. The proposed model results also outline the other baseline models in all key metrics, as presented in Table 13.

**Table 13:** Comparison of Performance Evaluation of Different Models on Test Set.

S. No.	Model	Testing Accuracy	Testing Loss	Precision	Recall	F1-Score
1	VGG-16	0.7143	0.7507	0.73	0.69	0.69
2	VGG-19	0.6842	0.8022	0.71	0.68	0.67
3	Inception V3	0.5246	2.2176	0.57	0.54	0.52
4	ResNet-50	0.7366	0.6455	0.74	0.71	0.71
5	EfficientNet-B0	0.7321	0.7139	0.75	0.72	0.72
6	EfficientNet-B4	0.7054	0.8039	0.75	0.71	0.71
7	EfficientNet-B4 + Self-attention	0.8234	0.4339	0.83	0.82	0.82
8	AttnEffNet-B4 (Proposed) (EfficientNet-B4 with Self-attention and dense layers)	0.9447	0.1988	0.93	0.92	0.92

### Stratified five-fold cross-validation

Further, to validate the performance of the proposed AttnEffNet-B4, a stratified five-fold cross-validation procedure is employed, which ensures each fold maintains the overall target-class distribution. Hence, the procedure reduces bias, imparting a comprehensive view of actual model generalization in the imbalanced dataset. The experimental results were overwhelmingly in favor of the architecture’s novelty and considered worthy of recommendation. Over the five folds, the model displayed very consistent accuracy scores of 0.8428, 0.8670, 0.8970, 0.9288, and 0.9377 for folds one to five, respectively. The mean accuracy was 0.8947, with a low standard deviation of 0.0360, which shows that the model varied little in its performance with the change in data partition. Similarly, the Area Under the Receiver Operating Characteristic Curve (AUC) scores further confirmed the outstanding classification capability of AttnEffNet-B4, posting values of 0.9710, 0.9811, 0.9853, 0.9903, and 0.9911 across the respective folds. Apparent from these consistently high AUC values was the remarkable discriminating power and reliability of the model.

**Fig. 12:**
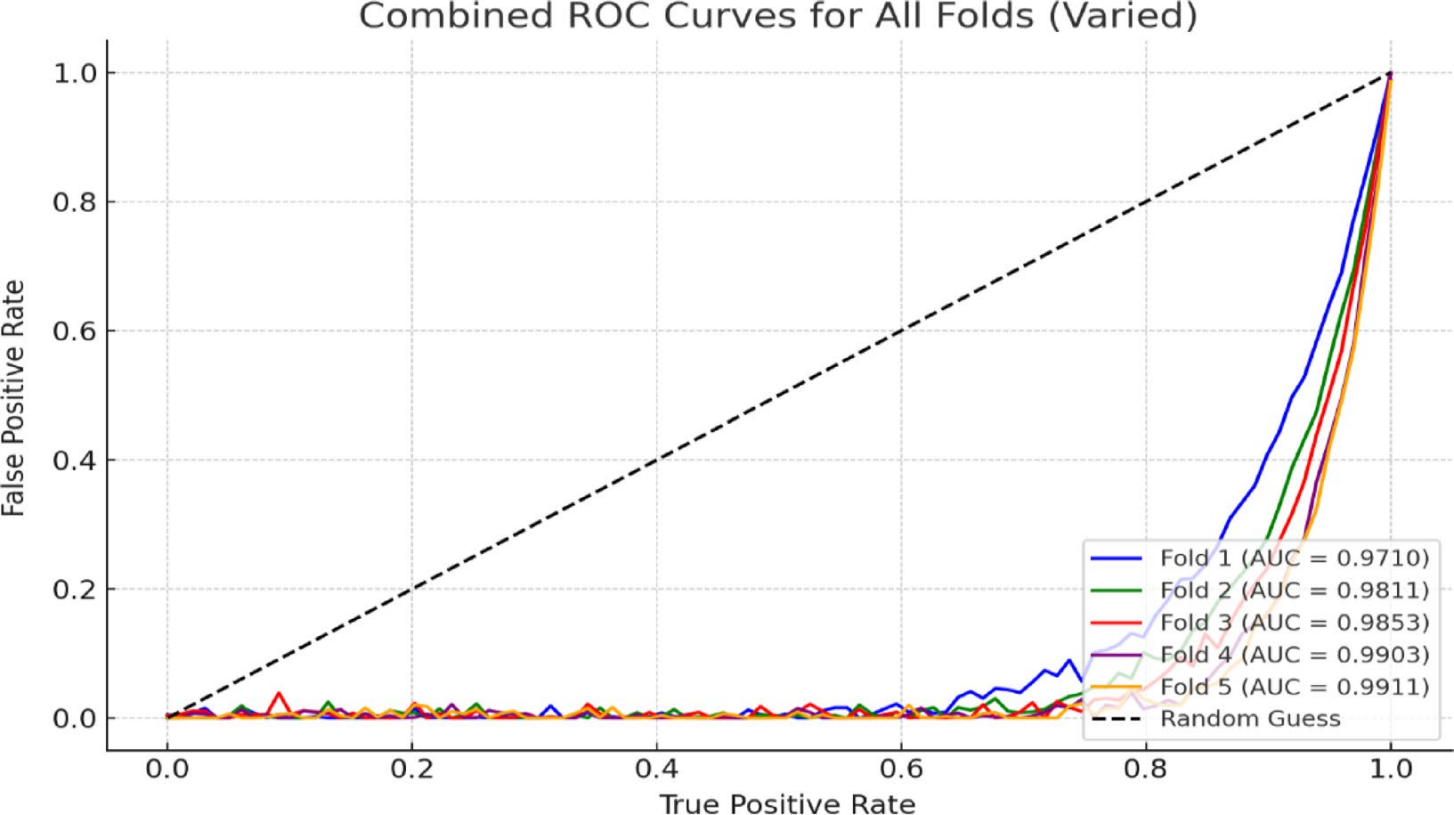
Combined ROC Curved for All Folds.

The combined ROC Plot, as illustrated in Fig. 12 combine all five folds together and provides further evidence of the effectiveness of the model. The curves show that the true TP rate is still high at different levels and clearly above the random level. These clearly solidify the argument in favor of this novel approach, which can thus be expected to render very accurate and reliable predictions in the intended application domain.

Table 14 represents the sustainability metrics of the proposed AttnEffNet-B4 model.

**Table 14:** Sustainability Metrics of AttnEffNet-B4.

SDG	Model Contribution	Impact
SDG-3: Good Health and Well-Being	The proposed model aids in early and accurate disease diagnosis.	Improved patient outcomes, help in reducing the burden of a disease diagnosis, and also impact the timely decision-making.
	It facilitates proactive patient care.	
	Under-resourced area access can be improved.	
SDG-9: Industry, Innovation, and Infrastructure	This AI-driven approach helps to reduce the need for diagnostic labs for individual diseases.	Cost-Effective and Scalability in Healthcare.
	Promotes telemedicine and digital health.	
SDG-10: Reduced Inequalities	Ensures that everyone has equal access to high-quality healthcare by promoting equitable and inclusive healthcare services for individuals in all areas.	Equitable access to quality healthcare services.

### Limitations of the proposed AttnEffNet-B4

Although the suggested AttnEffNet-B4 model is highly efficient in terms of its effectiveness in various and different disease types, there are some shortcomings that must be acknowledged. The model presents a moderate growth in the computational needs over baseline architectures that can require the use of a GPU-enabled environment in order to ensure optimal performance. But this trade-off allows for representing features better, as well as making classification more accurate. Also, despite the role of attention mechanisms to enhance the discrimination of features, the model does not explicitly consider some uncertainty estimation, which may be relevant in clinical decision support systems. The present analysis is done on a curated dataset, and additional validation on larger and multi-centric datasets would enhance the generalizability of the model to a wide variety of clinical settings.

## Conclusion and future scope

Healthcare systems globally face a growing challenge due to various factors such as the environment, our food habits, a tremendous rise in the patient load, and a limited doctor-to-patient ratio. The shortage of medical professionals, among other factors, usually leads to late diagnosis and poor care of the patient. This study addressed these issues by introducing a DL-based diagnostic model, namely AttnEffNet-B4, based on EfficientNet-B4, which employs self-attention, enabling accurate prediction of four critical diseases affecting the skin, brain, eye, and lung. The results of the experiments have shown that the proposed model has a high accuracy of up to 97.69% on the training data and 94.47% on the testing data in all the disease types, which outperformed the other existing CNN models. An analysis of the proposed model is also done with some other performance metrics like precision, recall, and F1 score. This study demonstrates that the use of self-attention increases the feature extraction, concentrating on the relevant regions of medical images, which results in improved performance in the case of a diagnosis. The results section depicts this with the assistance of heat maps and model performance evaluation metrics, as well as the k-fold analysis. In addition to that, it is concluded that the methodology saves significantly on the time of diagnosis, and it is a useful tool that would help medical professionals detect the disease at its early stages and plan treatment. The prospect that MltD diagnosis will be applied to automated comprehensive healthcare systems in the future is a great advantage. It is possible to make the work of clinics simpler, reduce their reliance on many specialized models, and make them more accessible, particularly in underprivileged or rural settings, with a single AI-driven diagnostic solution that can detect multiple diseases.

Although with positive outcomes, the proposed model can be developed and enhanced in several ways in the future. The integration of multimodal data, including laboratory findings and medical history of the patient, to a greater extent makes it possible to more thoroughly diagnose and apply it to a broader spectrum of diseases. In addition, future research can be made more effective by describing certain applications, including inference on low-power GPU usage in telemedicine services, a lightweight EfficientNet backbone that allows the introduction of the model into the electronic health record environment, and real-time deployment plans in cloud-assisted diagnostic models. Further efforts will also be needed for the improvement of computational efficiency by means of model optimization strategies like pruning and quantization, and the inclusion of explainable AI. The accessibility gap in healthcare will help physicians make quicker and more accurate judgments, and eventually enhance patient outcomes by using AI-driven diagnostic tools. Overall, these developments can enhance accessibility, help clinicians make the right decisions as quickly as possible, and lead to the further introduction of AI-driven solutions in healthcare.

## Data Availability

The dataset analyzed during the current study is publicly available in the Kaggle repository: https://www.kaggle.com/datasets/praneshkumarm/multidiseasedataset/.
